# *BMC Geriatrics* reviewer acknowledgement 2014

**DOI:** 10.1186/s12877-015-0009-0

**Published:** 2015-03-07

**Authors:** Giulia Mangiameli

**Affiliations:** BioMed Central, Floor 6, 236 Gray’s Inn Road, London, WC1X 8HB UK

## Abstract

The editors of BMC Geriatrics would like to thank all our reviewers who have contributed to the journal in Volume 14 (2014).

**Wilco Achterberg**

Netherlands

**Francisco Acurcio**

Brazil

**Kristina Akesson**

Sweden

**Theresa Allain**

Malawi

**Fred Andersen**

Norway

**Melissa K. Andrew**

Canada

**Hidenori Arai**

Japan

**Glenn Arendts**

Australia

**Joshua Armstrong**

Canada

**Benoit Arsenault**

Netherlands

**Erik Augustson**

USA

**Xue Bai Hong**

Kong

**Gillian Barry**

UK

**Peter Bartlett**

UK

**Stephane Baudry**

Belgium

**Marla Beauchamp**

USA

**Clemens Becker**

Germany

**Giuseppe Bellelli**

Italy

**Joel Belmin**

France

**Melissa Benton**

USA

**Rainer Beurskens**

Germany

**Kamaldeep Bhui**

UK

**Mark Bicket**

USA

**Ross Black**

Australia

**Florian Bleibler**

Germany

**Thomas Bodenheimer**

USA

**Froukje Boersma**

Netherlands

**Erin Bouldin**

USA

**Elisabeth Boulton**

UK

**Susan Bowles**

Canada

**Michael Brach**

Germany

**Elizabeth Breeze**

UK

**Emily Brogan**

Australia

**Rebecca Brown**

USA

**Ana-Maria Buga**

Germany

**Frances Bunn**

UK

**Matthew Bush**

USA

**Bianca Maria Buurman**

Netherlands

**Jeff Caird**

Canada

**Philip Calder**

UK

**Simone Caljouw**

Netherlands

**Michele Callisaya**

Australia

**Marcela Carrasco**

Chile

**Calogero Caruso**

Italy

**Robin Casten**

USA

**Carlo Cervellati**

Italy

**Ding-Cheng Chan**

Taiwan

**Yoon-Seok Chang**

South Korea

**Satabdi Chatterje**

USA

**Pui Hing Chau**

Hong Kong

**Huey-Tzy Chen**

Taiwan

**Huashuai Chen**

USA

**Chung-Hwan Chen**

Taiwan

**Liang-Kung Chen**

Taiwan

**Shuai Chen**

USA

**Yu-Chun Chen**

Taiwan

**Sheung-Tak Cheng**

Hong Kong

**Karen Siu Lan Cheung**

Hong Kong

**Mei Sian Chong**

Singapore

**Kee-Lee Chou**

China

**Vivienne Chuter**

Australia

**Arrigo Fg Cicero**

Italy

**Andrew Claus**

Australia

**Antonio Coca**

Spain

**Shameka Cody-Humphrey**

USA

**Mauro Colombo**

Italy

**Simon Conroy**

UK

**Rachel Cooper**

UK

**Bernard Cortet**

France

**Michael Crary**

USA

**Marilyn Cree**

Canada

**Nathan Davies**

UK

**Jennifer Davis**

Canada

**Piers Dawes**

UK

**Eling D. De Bruin**

Switzerland

**Stefanie De Buyser**

Belgium

**Leen De Coninck**

Belgium

**Lieven De Maesschalck**

Belgium

**Cesar De Oliveira**

UK

**Francesca De Terlizzi**

Italy

**Marjolein De Vugt**

Netherlands

**Anja Declercq**

Belgium

**Kim Delbaere**

Australia

**Liesbet Demarré**

Belgium

**Mario Di Napoli**

Italy

**Martin Dichter**

Germany

**Lars Donath**

Switzerland

**Wendy Duggleby**

Canada

**Eamonn Eeles**

Australia

**Thorlene Egerton**

Norway

**Maha El Gaafary**

Egypt

**Grahame Elder**

Australia

**Coralie English**

Australia

**Mark Fagan**

Norway

**Rui Felgueiras**

Portugal

**Steffen Fieuws**

Netherlands

**Joseph Finkelstein**

USA

**Trine Elisabeth Finnes**

Norway

**Thomas Fischer**

Germany

**George Fitchett**

USA

**Johan Flamaing**

Belgium

**Leon Flicker**

Australia

**Scott Forbes**

Canada

**Kristian Steen Frederiksen**

Denmark

**Ellen Freiberger**

Germany

**Michael Freitag**

Germany

**Yoshinori Fujiwara**

Japan

**Yoshiharu Fukuda**

Japan

**Carlo Gabelli**

Italy

**Michelle Gagnon**

Canada

**Josep Garre-Olmo**

Spain

**Nick Gebruers**

Belgium

**Suzanne Geerlings**

Netherlands

**Rebecca Genoe**

Canada

**Clarissa Giebel**

UK

**Timothy Girard**

USA

**Andrea Giusti**

Italy

**Danijela Gnjidic**

Australia

**Fernando Goglia**

Italy

**Adam L Gordon**

UK

**Urs Granacher**

Germany

**William B. Grant**

USA

**Amanda Grenier**

Canada

**Meredith Gresham**

Australia

**Yves Gschwind**

Australia

**Danan Gu**

USA

**Antonio Guaita**

Italy

**Valeri Guajardo**

Brazil

**Maelenn Guerchet**

UK

**Jason Guertin**

Canada

**Man Guo**

USA

**Thora Hafsteinsdottir**

Netherlands

**Klaus Hager**

Germany

**Ruud Halfens**

Netherlands

**Christine Hartmann**

USA

**Yvonne Heerkens**

Netherlands

**Stephan Heinzel**

Germany

**Jorunn Helbostad**

Norway

**Francois Herrmann**

Switzerland

**Mira Hidajat**

USA

**Andy Hau Yan Ho**

Hong Kong

**Lee Hooper**

UK

**Frances Horgan**

Ireland

**Fei-Yuan Hsiao**

Taiwan

**Kuangshi Huang**

China

**Paulette Hunter**

Canada

**Bettina Husebo**

Norway

**Valeria Isella**

Italy

**Tatsuro Ishizaki**

Japan

**Susan Jaglal**

Canada

**Mary Janevic**

USA

**Barbara Jefferis**

UK

**Ruth Jepson**

UK

**Antony Johansen**

UK

**Lena Johansson**

Sweden

**Becca Jordre**

USA

**Anthony Jorm**

Australia

**Olivia Kada**

Austria

**Pekka Kannus**

Finland

**Gertrudis I.J.M. Kempen**

Netherlands

**Marie-Jeanne Kergoat**

Canada

**Ngaire Kerse**

New Zealand

**Rahul Khanna**

USA

**Mijin Kim**

South Korea

**Naruki Kitano**

Japan

**Taro Kojima**

Japan

**Daniel Kopf**

Germany

**Goran Koracevic**

Serbia

**Rumi Kozakai**

Japan

**Reto W. Kressig**

Switzerland

**Morten Tange Kristensen**

Denmark

**Edeltraut Kröger**

Netherlands

**Vilai Kuptniratsaikul**

Thailand

**Alexander Kurz**

Germany

**Linda Lam**

Hong Kong

**Sarah Lamb**

UK

**Claudine Lamoth**

Netherlands

**Carole-Lynne Le Navenec**

Canada

**Jean Yves Le Reste**

France

**Sandra Lefort**

Canada

**Anja Leist**

Luxembourg

**Melanie Lesinski**

Germany

**Joan Leung**

Australia

**Melanie Li**

Taiwan

**Yi Li**

China

**Tianyuan Li**

Hong Kong

**Jae-Young Lim**

South Korea

**Ulrich Lindemann**

Germany

**Richard Lindley**

Australia

**Tsan-Hon Liou**

Taiwan

**Benjamin Liptzin**

USA

**Melanie Logue**

USA

**Carl Lombard**

South Africa

**Vivian W. Q. Lou**

Hong Kong

**Lee-Fay Low**

Australia

**Lisa Low**

Hong Kong

**Nan Lu**

China

**Elena Lucchi**

Italy

**Terry Lum**

Hong Kong

**Duncan Macfarlane**

Hong Kong

**Shylie Mackintosh**

Australia

**Davide Malatesta**

Switzerland

**Daniel Malone**

USA

**Alayne Markland**

USA

**Ana Isabel Marques**

Portugal

**Manuel Martin-Carrasco**

Spain

**Nicolás Martínez-Velilla**

Spain

**Emanuele Marzetti**

Italy

**Roy Mathew**

USA

**Colleen Maxwell**

Canada

**Cathal Mccrory**

Ireland

**Jane Mccusker**

Canada

**Katherine S Mcgilton**

Canada

**Patrizia Mecocci**

Italy

**Graydon Meneilly**

Canada

**Gabriele Meyer**

Germany

**Laura Middleton**

Canada

**Amanda Miller Amberber**

Australia

**Veronica Mocanu**

Romania

**Roberto Monastero**

Italy

**Kirsten Moore**

Australia

**Nicholas Moore**

France

**Rodrigo Moreira**

Brazil

**Mario Luca Morieri**

Italy

**Cristiano Moura**

Canada

**Martin Mueller**

Germany

**Judy Muller-Delp**

USA

**Nanette Mutrie**

UK

**Koutatsu Nagai**

Japan

**Alicia Nelson**

USA

**Karin Neufeld**

USA

**Louisa Ng**

Australia

**Tuan Nguyen**

Australia

**Anna Nieboer**

Netherlands

**Akitoshi Nishishita**

Japan

**Shwe Zin Nyunt**

Singapore

**Christiane Oedekoven**

UK

**Nelly Oelke**

Canada

**Yoshiro Okubo**

Japan

**Marcel Olde Rikkert**

Netherlands

**Jacilyn Olson**

USA

**Desmond O'Neill**

Ireland

**Bregje D Onwuteaka-Philipsen**

Netherlands

**Carlos Orces**

USA

**Matthew Parsons**

New Zealand

**Sahdia Parveen**

UK

**Anita Patel**

UK

**Eyvind Paulssen**

Norway

**Juan D. Pedrera-Zamorano**

Spain

**Carlo Pedrolli**

Italy

**Nancye May Peel**

Australia

**Geeske Peeters**

Australia

**Eugen-Bogdan Petcu**

Australia

**Genevieve Pham Kanter**

USA

**Evelien Pijpers**

Netherlands

**Giulio Pioli**

Italy

**Jenny Ploeg**

Canada

**Kristina Radinovic**

Serbia

**Parvin Rahnama**

Iran

**Kilian Rapp**

Germany

**Michael Rapp**

Germany

**Irene Maeve Rea**

UK

**Bjorn Rosengren**

Sweden

**Renzo Rozzini**

Italy

**Alessandro Rubinacci**

Italy

**Jorge Ruivo**

Portugal

**Tom Russ**

UK

**David Russell**

USA

**Liv Wergeland Sorbye**

Norway

**Mahshid Saghazadeh**

Japan

**Zhu Sai Nan**

China

**Fabio Salvi**

Italy

**Raluca Elena Sandu Vintilescu**

Romania

**Gill Sarre**

UK

**Kai-Uwe Saum**

Germany

**Ingmar Schäfer**

Germany

**Lori Schindel Martin**

Canada

**Daniel Schoene**

Australia

**Birgitte Jjm Schoenmakers**

Belgium

**Nadja Schott**

Germany

**Rene Schwendimann**

Switzerland

**Michael Schwenk**

USA

**David Scott**

Australia

**Carlo Serrati**

Italy

**Hyehyung Shin**

South Korea

**Shoji Shinkai**

Japan

**Hiromi Shiraishi**

Japan

**Joanie Sims-Gould**

Canada

**Sarianna Sipilä**

Finland

**Martin Smalbrugge**

Netherlands

**Alex Smithson**

Spain

**Rafael Solana**

Spain

**Stanislaw Solnik**

USA

**Yuki Soma**

Japan

**Yuting Song**

USA

**Vincent Staggs**

USA

**Els Steeman**

Belgium

**Magnus Stenhagen**

Sweden

**Joanna Stewart**

New Zealand

**Junko Sugama**

Japan

**Caroline Sutcliffe**

UK

**Michele Talley**

USA

**Nanako Tamiya**

Japan

**Rajesh Tampi**

USA

**Cara Tannenbaum**

Canada

**Kristin Taraldsen**

Norway

**Der-Cherng Tarng**

Taiwan

**Hans Thulesius**

Sweden

**Chris Todd**

UK

**Christophe Trivalle**

France

**Taishi Tsuji**

Finland

**Kenji Tsunoda**

Japan

**Anthony Tuckett**

Australia

**Maarit Katariina Valtonen**

Finland

**Marian A.E. Van Bokhorst - De Van Der Schueren**

Netherlands

**Lieve Van Den Block**

Belgium

**Marlise Van Eersel**

Netherlands

**Paula Van Wyk**

Canada

**David Vance**

USA

**Massimo Venturelli**

Italy

**Hilde Verbeek**

Netherlands

**Davide Liborio Vetrano**

Italy

**Renuka Visvanathan**

Australia

**Angela Vivanti**

Australia

**Stefano Volpato**

Italy

**Wolfgang Von Renteln-Kruse**

Germany

**Ake B. Wahlin**

Sweden

**Jingjy Wang**

Taiwan

**Pei-Ning Wang**

UK

**Christine Wann-Hansson**

Sweden

**Katherine Ward**

USA

**Rachel Ward**

USA

**Eiichi Watanabe**

Japan

**Joseph Wherton**

UK

**Julie Whitney**

UK

**Birgitt Wiese**

Germany

**Jacek M. Witkowski**

Poland

**Peter Wolf**

Switzerland

**Karin Wolf-Ostermann**

Germany

**Martin Wolkewitz**

Germany

**Bettina Wollesen**

Germany

**Gloria Wong**

Hong Kong

**Kuangnan Xiong**

USA

**Dongjuan Xu**

USA

**Fang Yang**

China

**Yen Kuang Yang**

Taiwan

**Charles W. Yates**

USA

**Dannii Yeung**

Hong Kong

**Abebaw, Mengistu Yohannes**

UK

**Jiyeong Yoon**

Japan

**Jieun Yoon**

Japan

**Andrea Zammit**

USA

**Laura Zannini**

Italy

**Astrid Zech**

Germany

**Fengyu Zhang**

USA

**Xin Zhang**

China

**Li Zhang**

China

**Quan Zhou**

China

**Haiyan Zhu**

USA

**Giovanni Zuliani**

Italy

